# Fulvic Acid Promotes the Reduction of Hexavalent Chromium by *Shewanella putrefaciens* via N-acylated-_L_-homoserine Lactones-Mediated Quorum Sensing

**DOI:** 10.3390/toxics13090708

**Published:** 2025-08-22

**Authors:** Xusheng Zheng, Xiaoyue Li, Yanping Liu, Guangqing Liu, Ziyi Yang, Dexun Zou

**Affiliations:** Department of Environmental Science & Engineering, Beijing University of Chemical Technology, Beijing 100029, China; zhengxusheng@mail.buct.edu.cn (X.Z.);

**Keywords:** microbial Cr(VI) reduction, fulvic acid, AQS, AHLs, quorum sensing

## Abstract

Extracellular electron transfer is crucial in the microbial reduction of hexavalent chromium [Cr(VI)], and N-acylated-_L_-homoserine lactones (AHLs) could accelerate this process. In this study, fulvic acid (FA) was used as an electron shuttle to enhance the microbial reduction process via stimulating extracellular electron transfer efficiency. Compared with 9,10-anthraquinone-2-sulfonic acid (AQS), FA had a stronger positive effect on Cr(VI) reduction by *S. putrefaciens*, showing the ability of stimulating *S. putrefaciens* to release AHLs. The concentrations of C6-HSL, C8-HSL and 3OC10-HSL increased by 11.79 ng/L, 19.82 ng/L and 3.01 ng/L after the addition of 2% FA. The bioinformation analysis indicated that AHLs could regulate the synthesis of electron shuttles by *S. putrefaciens*, such as riboflavin. And the addition of exogenous C6-HSL, C8-HSL, C10-HSL, C12-HSL and 3OC10-HSL increased the Cr(VI) reduction rates by 1.73%, 2.39%, 4.18%, 1.45% and 2.70%, because they could promote the release of riboflavin. It revealed a new pathway by which FA promoted microbial Cr(VI) reduction. This study also provides a novel approach for enhancing the microbial Cr(VI) reduction and a deeper understanding of the communication mechanism among microorganisms.

## 1. Introduction

Chromium (Cr), which is extensively utilized in multiple industries like electroplating, dye manufacturing and leather tanning, poses significant environmental pollution risks upon leakage [1,2]. In the environment, Cr primarily exists in two valent states: trivalent [Cr(III)] and hexavalent [Cr(VI)]. Cr(VI) is particularly concerning due to its high solubility, mobility [3,4], and associated carcinogenic, teratogenic and mutagenic effects [5,6]. Cr(III) exhibits lower mobility and toxicity [7]. Consequently, the primary remediation strategy involves reducing Cr(VI) to the less harmful Cr(III) form.

Among Cr(VI) remediation approaches, microbial remediation stands out as a promising technique owing to its environmental friendliness and cost-effectiveness [8,9]. In the microbial reduction process of Cr(VI), extracellular electron transfer (EET) offers distinct advantages over intracellular enzymes reduction, as it prevents Cr(VI) from entering cells and causing toxicity [10,11].

Electron shuttles could facilitate the EET between microorganisms and Cr(VI). Microorganisms reduce quinone groups in electron shuttles to hydroquinones, which are then re-oxidized to quinones by terminal electron acceptors such as Cr(VI), thereby promoting EET efficiency [12]. 9,10-anthraquinone-2-sulfonic acid (AQS) and fluvic acid (FA) are commonly used electron shuttles [13]. A previous study found that both the content of quinone groups and electron accepting capacities followed the order AQS > FA [14]. However, FA, a crucial humic substances component, offers additional advantages: it has little impact on the environment and contains diverse oxidizable/reducible functional groups (e.g., hydroxyls, phenols, quinones and carboxyls) that could also act as electron shuttles to facilitate EET [15,16]. Nevertheless, which electron shuttle exerts a stronger promoting effect on microbial Cr(VI) reduction remains unclear.

Quorum sensing (QS), a microbial communication mechanism [17], regulates the gene expression and the fluctuation in population density [18]. It involves the production and release of extracellular signal molecules. N-acylated-l-homoserine lactones (AHLs) are a type of signal molecules used by Gram-negative bacteria in QS [19]. Several studies demonstrated that AHLs-mediated QS could accelerate EET [17,20,21]. Interestingly, FA could also enhance the microbial communication by stimulating the release of AHLs [22].

Therefore, the role of FA played in the process of microbial Cr(VI) reduction was investigated. Specifically, the main objectives of this study were (1) to compare the effect of FA and AQS on the microbial Cr(VI) reduction; (2) to explore the promoting release effect of FA on AHLs; and (3) to investigate the effect of AHL-mediated QS on microbial Cr(VI) reduction.

## 2. Materials and Methods

### 2.1. Microorganisms and Chemicals

*Shewanella putrefaciens* CN32 was chosen for the microbial Cr(VI) reduction since it was a strain of iron reducing bacteria and could reduce Cr(VI) through extracellular electron transport [2,23,24]. It was purchased from the China Center for Type Culture Collection (CCTCC) and cultured aerobically in Luria-Bertani medium at 30 °C and 180 rpm in a constant-temperature incubator. Cells in exponential growth phase were harvested by centrifugation (3500 RCF, 10 min) and washed three times with anoxic 4-(2-hydroxy-ethyl)-1piperazineethanesulfonic acid (HEPES) buffer (30 mM, pH 7.0). The cells were resuspended in anoxic HEPES buffer in an anoxic chamber for further use.

The synthesis method of ferrihydrite based on Ryden [25], which involved titrating 0.4 M Fe(NO_3_)_3_·9H_2_O with 1 M NaOH to a pH of 7, continuously stirring during the titration process, then washing the resulting suspension with deionized water several times before drying it with a freeze dryer.

FA, seven typical AHLs (C6-HSL, C8-HSL, C10-HSL, C12-HSL, 3OC8-HSL, 3OC10-HSL and 3OC12-HSL) and acylase (>0.5 U/mg) were purchased from Macklin (Shanghai, China). Potassium dichromate was used as the source of Cr(VI) and was obtained from Sinopharm Chemical Reagent Co. (Shanghai, China). All chemicals used in this study were of reagent grade or higher.

### 2.2. Quorum Sensing Genes in S. putrefaciens

Quorum sensing genes were mined by referring to the quorum sensing database (QSDB) and methods constructed by Du et al. [26]. Specifically, the complete genome of *S. putrefaciens* was downloaded from National Center for Biotechnology Information (NCBI) database [26] and aligned to the QSDB using BLAST (2.16.0). A gene was considered as a potential QS gene if it had over 40% identity to at least one gene in QS database with the BLAST E-value below 10^−4^. And according to the types of signal molecules, QS genes were divided into 7 categories, which were AHLs, AIP, AI-2, PQS, DSF, cdi-GMP and Others.

### 2.3. Microbial Cr(VI) Reduction in the Presence of Electron Shuttle and AHLs

Batch experiments were performed in glass media bottles to investigate the effect of different electron shuttles and AHLs on the microbial Cr(VI) reduction (Table 1). All preparations were conducted in an anoxic glove box. Reactors were filled with 100 mL of deoxygenated 30 mM HEPES buffer0 (pH 7.0) containing various combinations of Cr(VI) (30 mM), ferrihydrite (2.0 g/L) and 1 mL bacterial suspension (OD600 = 2.0). Excess sodium lactate (10 mM final concentration) was filter sterilized as the electron donor.

Group 1: In the experiment of comparing the promoting effects of different electron shuttles on microbial Cr(VI) reduction, 0.2% (*w*/*w*) FA and AQS were added to reactors. Control reactors did not add any electron shuttles.

Group 2: In the experiment of the effect of FA and AQS on the release of AHLs, different concentrations (0.2% and 2.0%, *w*/*w*) of FA and AQS were added to reactors. Control reactors did not add any electron shuttles.

Group 3: In the experiment of the effect of AHLs on Cr(VI) reduction, N-hexanoyl-L-homoserine lactone (C6-HSL), N-octanoyl-L-homoserine lactone (C8-HSL), N-decanoyl-L-homoserine lactone (C10-HSL), N-dodecanoyl-L-homoserine lactone (C12-HSL), N-(3-oxooctanoyl)-DL-homoserine lactone (3OC8-HSL), N-(3-oxodecanoyl)-L-homoserine lactone (3OC10-HSL), N-(3-oxododecanoyl)-L-homoserine lactone (3OC12-HSL) and acylase were added to reactors with the final concentration of 500 nmol/L. Control reactors did not add any AHLs or acylase.

In both groups, reactors were sealed with parafilm and incubated in a constant temperature incubator at 30 °C, 150 rpm. All experiments were run in duplicate. After cell addition, samples were periodically removed with sterile needle and syringe inside the anoxic glove box to determine the concentration of Cr(VI).

### 2.4. Effect of AHLs on the Release of Riboflavin

Batch experiments were performed in 250 mL conical flasks, which contained 100 mL Luria-Bertani medium and 1 mL bacterial suspension (OD600 = 2.0). Seven types of AHLs (C6-HSL, C8-HSL, C10-HSL, C12-HSL, 3OC8-HSL, 3OC10-HSL and 3OC12-HSL) were added to reactors with the final concentration of 500 nmol/L. Control reactors did not add any AHLs. All experiments were run in duplicate and incubated in a constant temperature incubator at 30 °C, 150 rpm. After cell addition, samples were periodically removed with sterile needle and syringe. The supernatants were subjected to riboflavin measurements.

**Figure 1:**
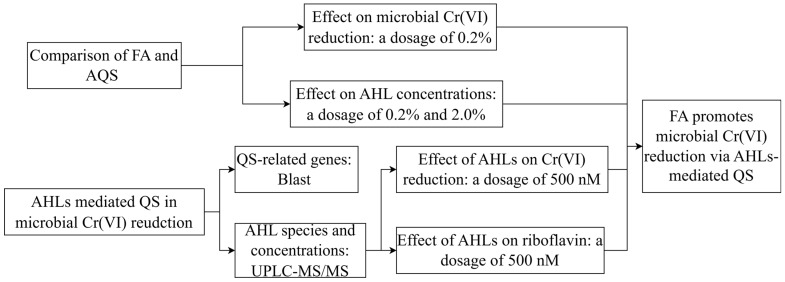
Schematic diagram of conducted experiments.

The schematic diagram of conducted experiments was shown in Figure 1.

### 2.5. Analytical and Statistical Methods

Aqueous Cr(VI) was qualified by the diphenylcarbazide method using a UV/Vis spectrophotometer at 540 nm [1]. AHLs was extracted from supernatants and concentrated by solid-phase extraction (SPE) using C18 columns (BKMAMALB, China) [27]. The concentrations of AHLs were analyzed by ultra-performance liquid chromatography (UPLC) equipped with an electrospray ionization source (ESI) [28]. The concentrations of riboflavin were determined using UPLC (Shimadzu, Japan) following a method reported previously [29].

The Cr(VI) reduction rate was modeled as pseudo-first-order and pseudo-second-order with respect to Cr(VI) concentration according to Formula (1) and (2). And Origin 2018 was used to draw all charts. All data are presented as mean ± SD. To determine significant differences between the experimental and control group means, a one-way analysis of variance (ANOVA) was conducted using IBM SPSS Statistics 26. When overall significant differences were identified, the least significant difference (LSD) post hoc test was subsequently applied to perform pairwise comparisons between specific group means. A significance level of *p* < 0.05 was used for all statistical tests.

(1) $$-\frac{d{\left[Cr\left(VI\right)\right]}_{t}}{dt}=k_{1}\times [Cr(VI)]$$

(2) $$\frac{1}{{\left[Cr\left(VI\right)\right]}_{t}}-\frac{1}{[{Cr\left(VI\right)]}_{0}}=k_{2}\times t$$

where *k*_1_ is the first-order rate constant (d^−1^); *k*_2_ is the second-order rate constant (d^−1^); [Cr(VI)]_t_ is the concentration of Cr(VI) at *t* time; [Cr(VI)]_0_ is the initial concentration of Cr(VI).

## 3. Results and Discussion

### 3.1. Effect of FA and AQS on Cr(VI) Reduction

**Figure 2:**
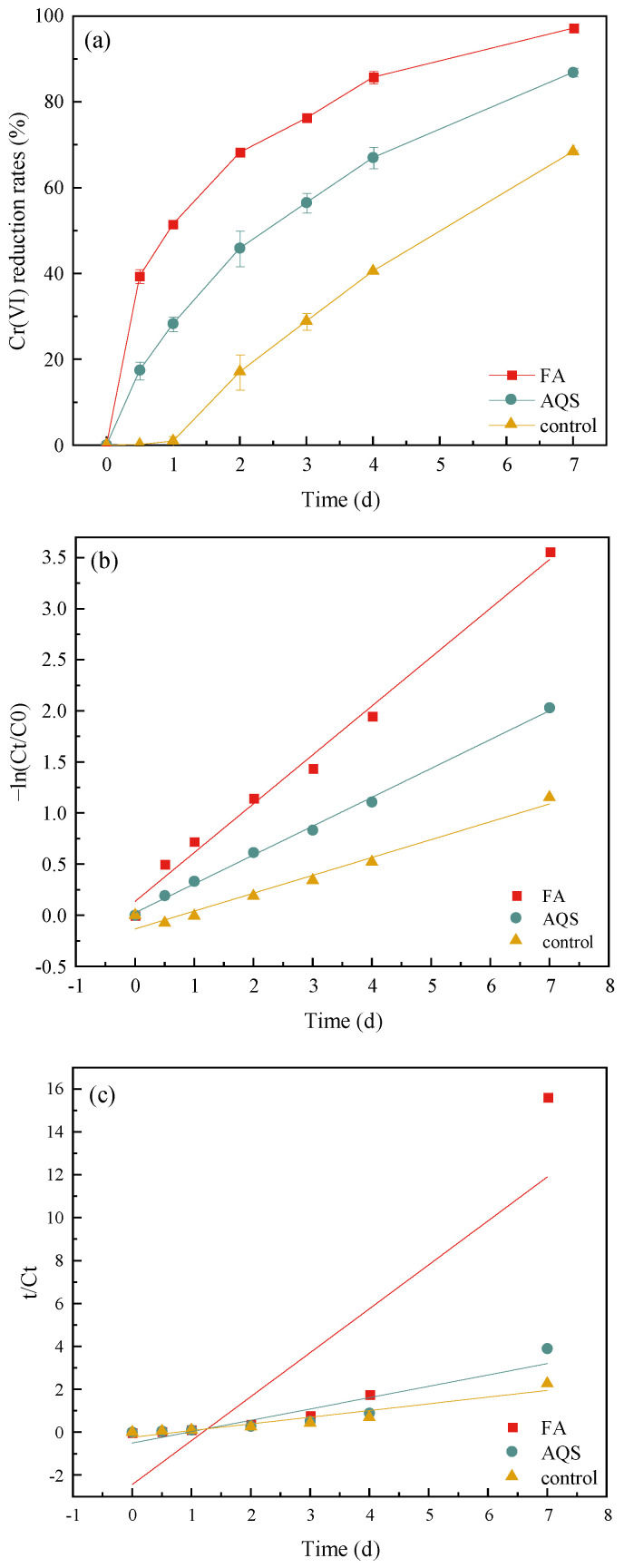
The effect of electron shuttles on Cr(VI) removal rate (**a**) and kinetic model fitting with respect to Cr(VI) concentration [pseudo-first-order (**b**) and pseudo-first-order (**c**)].

Without the addition of electron shuttle, the Cr(VI) reduction rates were low on day 0–1 (<1%, Figure 2a). Then the Cr(VI) reduction rates gradually increased and reached 68.42% on day 7. However, after adding FA and AQS, the Cr(VI) reduction rates reached 39.41% and 17.45% after operating 12 h, and then increased to 97.14% and 86.88% at the final state. It showed that electron shuttle could enhance the Cr(VI) reduction rate by *S. putrefaciens* effectively. In addition, the Cr(VI) reduction rates of the experimental group supplemented with FA were also consistently higher than that supplemented with AQS. The modeling results showed that the R^2^ of the pseudo-first-order model was greater that of the pseudo-second-order model, indicating that the variation of Cr(VI) was more consistent with the pseudo-first-order model (Table 2 and Figure 2b,c). The first-order rate constants for Cr(VI) reduction of experimental groups ranked as FA (0.478 d^−1^) > AQS (0.283 d^−1^) > control (0.175 d^−1^), suggesting that FA had a better promoting effect on Cr(VI) reduction rate by *S. putrefaciens* than AQS. However, Wu et al. found that AQS had a greater promoting effect on Fe(III) reduction by *Corynebacterium humireducens* MFC-5 than FA, because AQS showed the higher quinone group content and stronger electron accepting capacity than FA [14]. This demonstrated that FA may also be able to promote the Cr(VI) reduction by *S. putrefaciens* through other pathways such as QS.

### 3.2. Potential QS Genes in Genomes of S. putrefaciens

**Figure 3:**
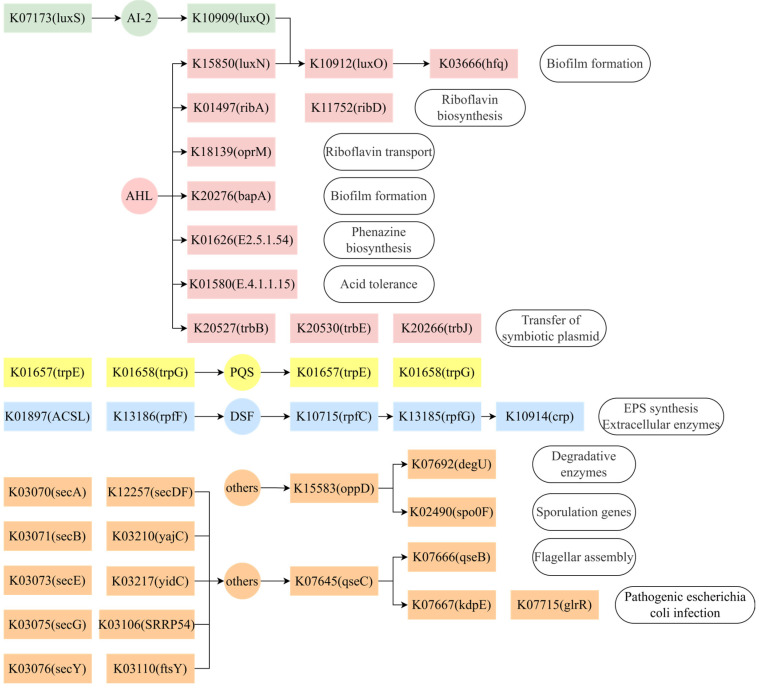
QS-related genes and regulated pathways in the genome of *S. putrefaciens* (Color represents pathways regulated by different auto-inducers).

There were 70 potential QS genes in the genomes of *S. putrefaciens*, which could be divided into seven categories based on the types of signal molecules (Table S1). Referring to the quorum sensing pathway (map02024) in the Kyoto Encyclopedia of Genes and Genomes (KEGG) database, it can be found that these QS genes could regulate various life activities of *S. putrefaciens* (Figure 3). There are several regulated pathways associated with AHL, such as biosynthesis and transport of riboflavin (ribA, ribD and oprM), biofilm formation (luxO, hfq and bapA), acid tolerance (E4.1.15), phenazine biosynthesis (E2.5.1.54), transfer of symbiotic plasmid (trbB, trbE and trbJ) and auto-inducer sensor kinase (luxN). In addition, an AHL-quenching gene (pvdQ) also existed.

There is also a pathway associated with DSF composed of ACSL, rpfF, rpfC, rpfG, crp and fhrR in *S. putrefaciens*, which could regulate extracellular polymeric substances (EPS synthesis) and extracellular enzymes. For the PQS-mediated QS system, *S. putrefaciens* contains auto-inducer synthase trpE and trpG. For the AI-2-mediated QS system, there are auto-inducer synthase (luxS), sensor kinase (luxQ) and regulated components (luxO and hfq). And they are related to biofilm formation.

In addition, other QS systems, such as degradative enzymes (secA, secB, secE, secG, secY, secDF, SRP 54, ftsY, yajC and degU) and sporulation genes (spo0F and oppD), which are mediated by the auto-inducers Phr, PapR and mature antimicrobial peptides, also exist in the genome of *S. putrefaciens*.

The above results indicate that there are multiple potential QS systems in *S. putrefaciens*, and various traits can be expressed under the regulation of different auto-inducers. The existence of AI-2-related QS genes indicates that they could communicate with other microorganisms. And the existence of an AHL-quenching gene speculated that *S. putrefaciens* may be regulated by AHL at an appropriate time; otherwise AHL would be quenched. What is more, the synthesis of riboflavin and phenazine, which could facilitate electron transfer from *S. putrefaciens* to iron oxides as electron shuttles [30,31], is regulated by AHLs. It suggests that AHL-mediated QS may be able to promote the Cr(VI) reduction by *S. putrefaciens*.

### 3.3. AHL Identification in the Process of the Microbial Cr(VI) Reduction

**Figure 4:**
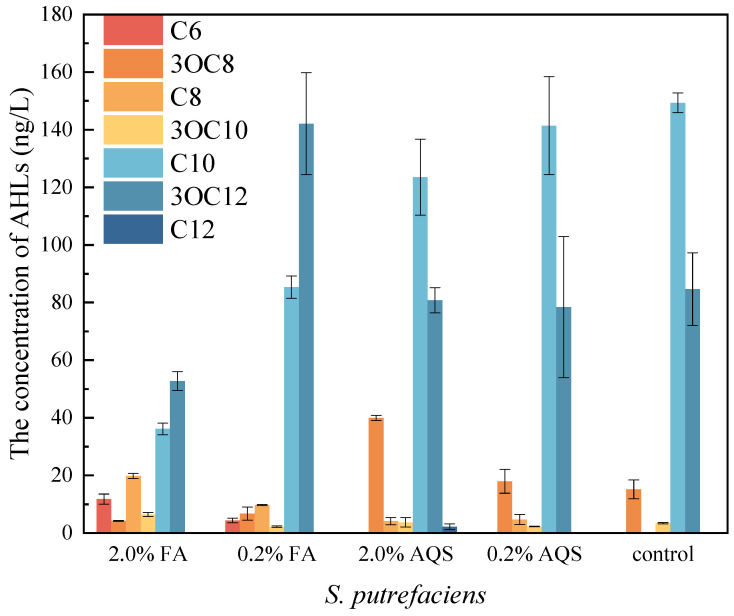
The effect of electron shuttles on AHLs concentrations.

In the process of Cr(VI) reduction by *S. putrefaciens*, the total concentration of AHLs was 252.65 ng/L, and there were multiple species of AHLs such as 3OC8-HSL, 3OC10-HSL, C10-HSL and 3OC12-HSL, with the concentrations of 15.17 ng/L, 3.44 ng/L, 149.38 ng/L and 84.66 ng/L (Figure 4). As the dosage of FA increased from 0.2% to 2.0%, two new types of AHLs emerged, i.e., C6-HSL and C8-HSL, with the concentrations of 11.79 ng/L and 19.82 ng/L, respectively. The concentration of 3OC10-HSL increased by 3.01 ng/L. However, the concentrations of 3OC8-HSL, C10-HSL and 3OC12-HSL decreased by 10.89 ng/L, 113.26 ng/L and 31.92 ng/L, leading to a decrease in the total concentration of AHLs. As the dosage of AQS increased from 0.2% to 2.0%, two new types of AHLs were obtained, i.e., C8-HSL (4.15 ng/L) and C12-HSL (2.18 ng/L). The concentration of 3OC8-HSL increased by 24.75 ng/L. However, the concentration of C10-HSL and 3OC12-HSL decreased by 25.88 ng/L and 3.85 ng/L, resulting in the weak increment of 1.66 ng/L.

These results showed that AHL-mediated QS existed in the Cr(VI) reduction by *S. putrefaciens*. In this process, FA could promote the release of C6-HSL, C8-HSL and 3OC10-HSL by *S. putrefaciens*, and AQS could promote the release of C8-HSL, 3OC8-HSL and C12-HSL. The reason for the difference may be that there were various other functional groups besides quinone group in FA, which could also exert influence on QS.

### 3.4. Effect of Exogenous AHLs on the Microbial Cr(VI) Reduction

**Figure 5:**
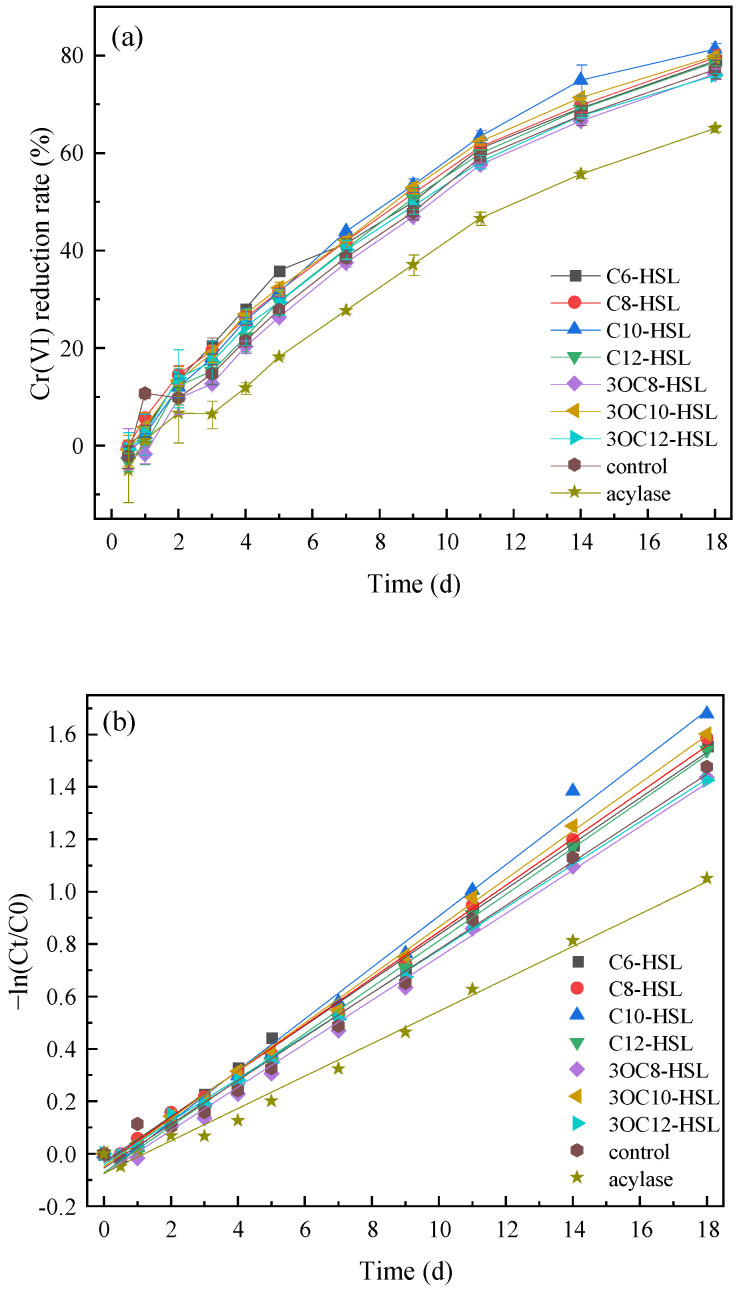

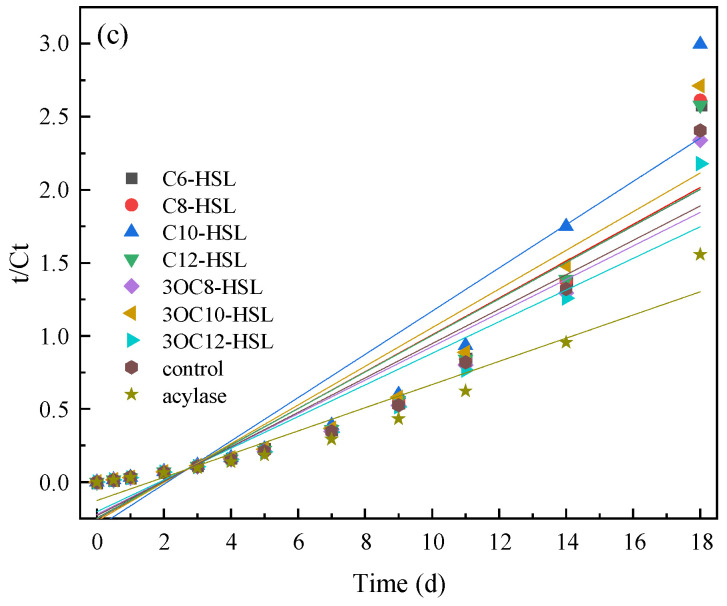
The effect of AHLs on Cr(VI) removal rate (**a**) and kinetic model fitting with respect to Cr(VI) concentration [pseudo-first-order (**b**) and pseudo-first-order (**c**)].

Acylase can separate the homoserine lactone ring and acyl side chains to generate fatty acids and homoserine lactones, thereby deactivating AHLs [32]. The Cr(VI) reduction rates via *S. putrefaciens* were significantly inhibited after adding acylase since the 3rd day, and the difference with the control group kept increasing to 12.10% on the 18th day (Figure 5a). After the addition of AHLs, the Cr(VI) reduction rates of all experimental groups on the 1st day were lower than that of the control group, with the decrement of 5.02–12.47%. From the 4th day, the Cr(VI) reduction rates of the experimental groups supplemented with C10-HSL and 3OC10-HSL were significantly higher than that of the control group (*p* < 0.05), and the maximum promoting effects were 7.24% and 5.00%. The addition of C6-HSL only significantly increased (*p* < 0.05) the Cr(VI) reduction rates on the 4th and 5th day, with the maximum increment of 8.06%. And the addition of C8-HSL significantly increased (*p* < 0.05) the Cr(VI) reduction rates from the 4th to 9th day, with the maximum increment of 4.69%. The modeling results showed that the R^2^ of the pseudo-first-order model was greater that of the pseudo-second-order model, indicating that the variation of Cr(VI) was more consistent with the pseudo-first-order model (Table 2, Figure 5b,c). The first-order rate constants for Cr(VI) reduction of experimental groups supplemented with C6-HSL, C8-HSL, C10-HSL, C12-HSL and 3OC10-HSL were higher than that of the control group, while those of experimental groups supplemented with 3OC8-HSL, 3OC12-HSL and acylase were lower than that of the control group. The above results indicated that C10-HSL and 3OC10-HSL could accelerate the reduction of Cr(VI) by *S. puterfaciens* in the middle and later stages of the experiment, and C6-HSL and C8-HSL could only promote the Cr(VI) reduction in the middle stage.

### 3.5. Functional Analysis of FA

**Figure 6:**
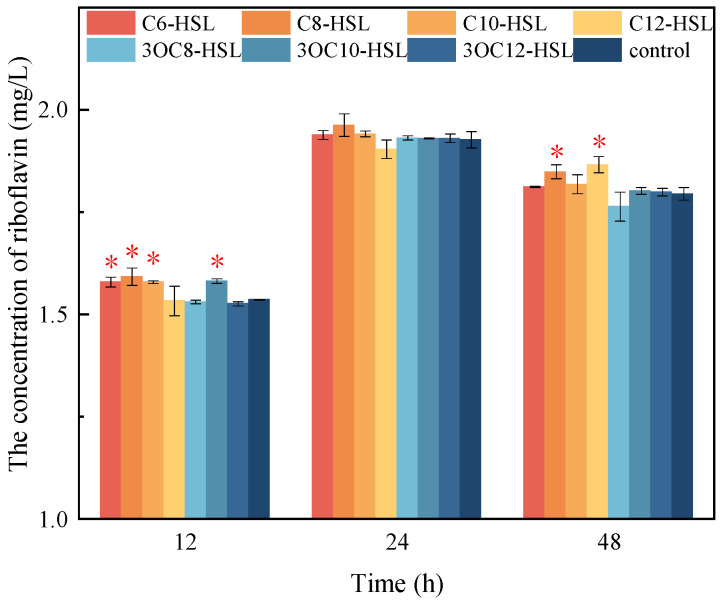
The effect of AHLs on the concentration of riboflavin (* above the columns represents a significant difference between the group with control group when *p* < 0.05).

*Shewanella* could secrete riboflavin as the electron shuttle to accelerate the extracellular electron transfer. After the addition of C6-HSL, C8-HSL, C10-HSL and 3OC10-HSL, the concentration of riboflavin significantly increased by 0.043 mg/L, 0.056 mg/L, 0.043 mg/L and 0.046 mg/L at 12 h (Figure 6). However, only the addition of C8-HSL and C12-HSL significantly increased the concentration of riboflavin by 0.054 mg/L and 0.071 mg/L at 48 h. It indicated that C6-HSL, C8-HSL, C10-HSL and 3OC10-HSL could promote the release of riboflavin in the early stage of experiment, while C8-HSL and C12-HSL could promote the release of riboflavin in the later stage.

At present, several studies have revealed that AHLs could accelerate electron transfer by promoting the secretion of electron shuttles. During the biochemical process, C4-HSL and 3OC6-HSL could increase the content of humic acid in EPS, thereby increasing the efficiency of bioreactions [33,34]. What is more, when AHL synthase was overexpressed in the strains, phenazines were produced as electron shuttles [35]. Chen et al. found that the expression of riboflavin synthase increased after the addition of AHLs [36]. In this paper, it was also confirmed from another perspective by the effect of AHLs on the concentrations of riboflavin.

**Figure 7:**
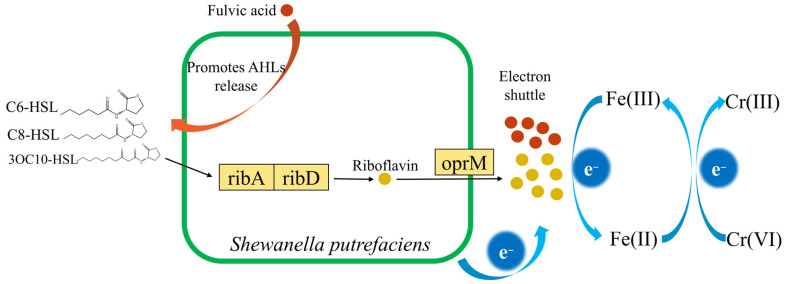
Mechanism of FA promoting the Cr(VI) reduction by *S. putrefaciens*.

Based on the above results, the analysis of FA promoting the Cr(VI) reduction by *S. putrefaciens* is speculated as follows (Figure 7): FA could stimulate *S. putrefaciens* to release C6-HSL, C8-HSL and 3OC10-HSL. These three AHLs in turn could regulate phenazine synthesis genes (E2.5.1.54), riboflavin biosynthesis genes (ribA and ribD) and riboflavin transport genes (oprM). The generated riboflavin, as well as FA, acted as electron shuttles to accelerate the Cr(VI) reduction by *S. putrefaciens*. It is precisely for this reason that, compared with AQS, FA could still have a better enhancing effect on Cr(VI) reduction by *S. putrefaciens* even with lower electron accepting capacity and content of quinone groups. And the promoted release of AHLs by FA is attributed to the various functional groups it contains other than quinone groups.

## 4. Conclusions

In this study, the role FA played in the process of microbial Cr(VI) reduction by *S. putrefaciens* was investigated. Compared with AQS, FA had better Cr(VI) reduction efficiency in the case of lower electron accepting capacity and fewer quinone groups, because FA stimulated the release of C6-HSL, C8-HSL and 3OC10-HSL. There were various potential QS systems in *S. putrefaciens*, and AHLs could regulate the synthesis of electron shuttles. C6-HSL, C8-HSL, C10-HSL, C12-HSL and 3OC10-HSL could promote the release of riboflavin and accelerate the microbial Cr(VI) reduction. It revealed that FA could facilitate microbial Cr(VI) reduction by stimulating the release of AHLs. This study provides a novel approach for enhancing the microbial Cr(VI) reduction, and further improves the understanding of the communication mechanism among microorganisms.

## Figures and Tables

**Table 1:** The additives in the reactors of each experimental group.

Additives		Electron Shuttle	AHLs
Group 1	1	0.2% FA	None
	2	0.2 AQS	
	3	None	
Group 2	1	0.2% FA	None
	2	2.0% FA	
	3	0.2% AQS	
	4	2.0% AQS	
	5	None	
Group 3	1	None	C6-HSL
	2		C8-HSL
	3		C10-HSL
	4		C12-HSL
	5		3OC8-HSL
	6		3OC10-HSL
	7		3OC12-HSL
	8		None
	9		AHLs quencher

**Table 2:** Summary of first-order and second-order rate constants for Cr(VI) reduction.

Electron Shuttle	AHLs	Time (d)	Pseudo-First-Order		Pseudo-Second-Order	
			*k*_1_ (d^−1^) ^a^	R^2 b^	*k*_2_(d^−1^) ^a^	R^2 b^
Group 1						
FA		0–7	0.478 ± 0.022	0.99	2.049 ± 0.519	0.71
AQS			0.283 ± 0.006	0.99	0.528 ± 0.100	0.82
control			0.175 ± 0.013	0.97	0.312 ± 0.046	0.88
Group 2						
	C6	0–18	0.087 ± 0.002	0.99	0.125 ± 0.015	0.87
	C8		0.089 ± 0.001	0.99	0.126 ± 0.015	0.86
	C10		0.098 ± 0.002	0.99	0.148 ± 0.018	0.87
	C12		0.089 ± 0.002	0.99	0.125 ± 0.015	0.87
	3OC8		0.083 ± 0.002	0.99	0.115 ± 0.013	0.88
	3OC10		0.091 ± 0.001	0.99	0.132 ± 0.015	0.87
	3OC12		0.082 ± 0.001	0.99	0.108 ± 0.011	0.89
	control		0.084 ± 0.002	0.99	0.118 ± 0.013	0.88
	acylase		0.062 ± 0.002	0.99	0.079 ± 0.007	0.93

^a^ The values represent the means ± standard error from duplicate samples. ^b^ R^2^ for regression of ln([Cr]t/[Cr]0) versus time of spike-period.

## Data Availability

Data will be made available upon request.

## Supplementary Materials

The following supporting information can be downloaded at: https://www.mdpi.com/article/10.3390/toxics13090708/s1, Table S1: Potential QS genes in the genome of *S. putrefaciens*.

## References

[1] Meng Y., Zhao Z., Burgos W.D., Li Y., Zhang B., Wang Y., Liu W., Sun L., Lin L., Luan F. (2018). Iron(III) Minerals and Anthraquinone-2,6-Disulfonate (AQDS) Synergistically Enhance Bioreduction of Hexavalent Chromium by Shewanella Oneidensis MR-1. Sci. Total Environ..

[2] Zhou T., Chen H., Guo X., Zhang J., Meng Y., Luan F. (2024). AQDS-Functionalized Biochar Enhances the Bioreduction of Cr(VI) by *Shewanella Putrefaciens* CN32. Chemosphere.

[3] Mo Z., Tai D., Zhang H., Shahab A. (2022). A Comprehensive Review on the Adsorption of Heavy Metals by Zeolite Imidazole Framework (ZIF-8) Based Nanocomposite in Water. Chem. Eng. J..

[4] Zeng H., Zeng H., Zhang H., Shahab A., Zhang K., Lu Y., Nabi I., Naseem F., Ullah H. (2021). Efficient Adsorption of Cr (VI) from Aqueous Environments by Phosphoric Acid Activated Eucalyptus Biochar. J. Clean. Prod..

[5] Pei Y., Tao C., Ling Z., Yu Z., Ji J., Khan A., Mamtimin T., Liu P., Li X. (2020). Exploring Novel Cr(VI) Remediation Genes for Cr(VI)-Contaminated Industrial Wastewater Treatment by Comparative Metatranscriptomics and Metagenomics. Sci. Total Environ..

[6] Qiu Y., Zhang Q., Gao B., Li M., Fan Z., Sang W., Hao H., Wei X. (2020). Removal Mechanisms of Cr(VI) and Cr(III) by Biochar Supported Nanosized Zero-Valent Iron: Synergy of Adsorption, Reduction and Transformation. Environ. Pollut..

[7] Ma L., Du Y., Chen S., Du D., Ye H., Zhang T.C. (2022). Highly Efficient Removal of Cr(VI) from Aqueous Solution by Pinecone Biochar Supported Nanoscale Zero-Valent Iron Coupling with *Shewanella oneidensis* MR-1. Chemosphere.

[8] Cheng Z.-H., Xiong J.-R., Min D., Cheng L., Liu D.-F., Li W.-W., Jin F., Yang M., Yu H.-Q. (2020). Promoting Bidirectional Extracellular Electron Transfer of Shewanella Oneidensis MR-1 for Hexavalent Chromium Reduction via Elevating Intracellular cAMP Level. Biotechnol. Bioeng..

[9] Liu T., Luo X., Wu Y., Reinfelder J.R., Yuan X., Li X., Chen D., Li F. (2020). Extracellular Electron Shuttling Mediated by Soluble C-Type Cytochromes Produced by *Shewanella oneidensis* MR-1. Environ. Sci. Technol..

[10] Cologgi D.L., Lampa-Pastirk S., Speers A.M., Kelly S.D., Reguera G. (2011). Extracellular Reduction of Uranium via Geobacter Conductive Pili as a Protective Cellular Mechanism. Proc. Natl. Acad. Sci. USA.

[11] Karthik C., Ramkumar V.S., Pugazhendhi A., Gopalakrishnan K., Arulselvi P.I. (2017). Biosorption and Biotransformation of Cr(VI) by Novel *Cellulosimicrobium Funkei* Strain AR6. J. Taiwan Inst. Chem. Eng..

[12] Li X., Liu L., Liu T., Yuan T., Zhang W., Li F., Zhou S., Li Y. (2013). Electron Transfer Capacity Dependence of Quinone-Mediated Fe(III) Reduction and Current Generation by Klebsiella Pneumoniae L17. Chemosphere.

[13] Liu W., Wu Y., Liu T., Li F., Dong H., Jing M. (2019). Influence of Incubation Temperature on 9,10-Anthraquinone-2-Sulfonate (AQS)-Mediated Extracellular Electron Transfer. Front. Microbiol..

[14] Wu C., Zhuang L., Zhou S., Yuan Y., Yuan T., Li F. (2013). Humic Substance-Mediated Reduction of Iron(III) Oxides and Degradation of 2,4-D by an Alkaliphilic Bacterium, *Corynebacterium humireducens* MFC-5. Microb. Biotechnol..

[15] Héry M., Rizoulis A., Sanguin H., Cooke D.A., Pancost R.D., Polya D.A., Lloyd J.R. (2015). Microbial Ecology of Arsenic-Mobilizing Cambodian Sediments: Lithological Controls Uncovered by Stable-Isotope Probing. Environ. Microbiol..

[16] Roden E.E., Kappler A., Bauer I., Jiang J., Paul A., Stoesser R., Konishi H., Xu H. (2010). Extracellular Electron Transfer through Microbial Reduction of Solid-Phase Humic Substances. Nat. Geosci..

[17] Liu W., Cai W., Ma A., Ren G., Li Z., Zhuang G., Wang A. (2015). Improvement of Bioelectrochemical Property and Energy Recovery by Acylhomoserine Lactones (AHLs) in Microbial Electrolysis Cells (MECs). J. Power Sources.

[18] Svenningsen S.L., Tu K.C., Bassler B.L. (2009). Gene Dosage Compensation Calibrates Four Regulatory RNAs to Control Vibrio Cholerae Quorum Sensing. EMBO J..

[19] Paquete C.M., Rosenbaum M.A., Bañeras L., Rotaru A.-E., Puig S. (2022). Let’s Chat: Communication between Electroactive Microorganisms. Bioresour. Technol..

[20] Venkataraman A., Rosenbaum M., Arends J.B.A., Halitschke R., Angenent L.T. (2010). Quorum Sensing Regulates Electric Current Generation of *Pseudomonas aeruginosa* PA14 in Bioelectrochemical Systems. Electrochem. Commun..

[21] Yong Y.-C., Yu Y.-Y., Yang Y., Liu J., Wang J.-Y., Song H. (2013). Enhancement of Extracellular Electron Transfer and Bioelectricity Output by Synthetic Porin. Biotechnol. Bioeng..

[22] Liu L., Ji M., Wang F., Tian Z., Wang T., Wang S., Wang S., Yan Z. (2020). Insight into the Short-Term Effect of Fulvic Acid on Nitrogen Removal Performance and N-Acylated-L-Homoserine Lactones (AHLs) Release in the Anammox System. Sci. Total Environ..

[23] Liu X., Chen M., Wang D., Du F., Xu N., Sun W., Han Z. (2024). Cr(VI) Removal during Cotransport of Nano-Iron-Particles Combined with Iron Sulfides in Groundwater: Effects of *D*. *vulgaris* and *S. putrefaciens*. J. Hazard. Mater..

[24] Zhang B., Jiao W. (2022). Biochar Facilitated Bacterial Reduction of Cr(VI) by *Shewanella putrefaciens* CN32: Pathways and Surface Characteristics. Environ. Res..

[25] Ryden J.C., Syers J.K., Tillman R.W. (1987). Inorganic Anion Sorption and Interactions with Phosphate Sorption by Hydrous Ferric Oxide Gel. J. Soil Sci..

[26] Du Q., Mu Q., Wu G. (2021). Metagenomic and Bioanalytical Insights into Quorum Sensing of Methanogens in Anaerobic Digestion Systems with or without the Addition of Conductive Filter. Sci. Total Environ..

[27] Li X., Fekete A., Englmann M., Götz C., Rothballer M., Frommberger M., Buddrus K., Fekete J., Cai C., Schröder P. (2006). Development and Application of a Method for the Analysis of *N*-Acylhomoserine Lactones by Solid-Phase Extraction and Ultra High Pressure Liquid Chromatography. J. Chromatogr. A.

[28] Hu H., He J., Liu J., Yu H., Zhang J. (2016). Biofilm Activity and Sludge Characteristics Affected by Exogenous N-Acyl Homoserine Lactones in Biofilm Reactors. Bioresour. Technol..

[29] Zhao J., Li F., Kong S., Chen T., Song H., Wang Z. (2023). Elongated Riboflavin-Producing Shewanella Oneidensis in a Hybrid Biofilm Boosts Extracellular Electron Transfer. Adv. Sci..

[30] Li F., Zhang B., Long X., Yu H., Shi S., You Z., Liu Q., Li C., Tang R., Wu S. (2025). Dynamic Synthesis and Transport of Phenazine-1-Carboxylic Acid to Boost Extracellular Electron Transfer Rate. Nat. Commun..

[31] Yu Y., Li A., Fan S.-Q., Zhao H.-P. (2024). Biogenic Amorphous FeOOH Activated Additional Intracellular Electron Flow Pathways for Accelerating Reductive Dechlorination of Tetrachloroethylene. Water Res..

[32] McBride S.G., Strickland M.S. (2019). Quorum Sensing Modulates Microbial Efficiency by Regulating Bacterial Investment in Nutrient Acquisition Enzymes. Soil Biol. Biochem..

[33] Fu W., Li M., Dang W., Zhu K., Chen G., Zhang J., Wang S., Guo Y., Wang Z. (2022). Study on the Mechanism of Inhibiting the Calcification of Anaerobic Granular Sludge Induced by the Addition of Trace Signal Molecule (3O-C6-HSL). Bioresour. Technol..

[34] Li Y., Fan C., Liu L., Zhai X., Zang B., Li Y.-Y., Chen R. (2025). Biochar-Induced Quorum Sensing Enhances Methane Production by Strengthening Direct Interspecies Electron Transfer. Bioresour. Technol..

[35] Yong Y.-C., Yu Y.-Y., Li C.-M., Zhong J.-J., Song H. (2011). Bioelectricity Enhancement via Overexpression of Quorum Sensing System in *Pseudomonas aeruginosa*-Inoculated Microbial Fuel Cells. Biosens. Bioelectron..

[36] Chen L., Zhang P., Li Y., Liang J., Zhang G. (2024). Genome-Centric Metagenomic Analysis Reveals Mechanisms of Quorum Sensing Promoting Anaerobic Digestion under Sulfide Stress: Syntrophic Metabolism and Microbial Self-Adaptation. Sci. Total Environ..

