# Supplementary material for: Fulvic Acid Promotes the Reduction of Hexavalent Chromium by Shewanella putrefaciens via N-acylated-L-homoserine Lactones-Mediated Quorum Sensing
Source: Toxics. 2025 Aug 22;13(9):708. doi: 10.3390/toxics13090708 (PMC12473820; doi:10.3390/toxics13090708)
Supplement: Supplementary file 1 [file toxics-13-00708-s001.zip › toxics-3794341-supplementary.pdf]

Supplementary Materials

Supplementary Table

Table S1 Potential QS genes in the genome of *S. putrefaciens*

QS group	KO.	QS gene	Identify	E value
AHL	K03666	hfq	100.00	3.13×10^{-62}
AHL	K01497	ribA	100.00	3.74×10^{-154}
AHL	K01626	E2.5.1.54, aroF, aroG, aroH	100.00	0
AHL	K07116	pvdQ, quiP	99.88	0
AHL	K01580	E4.1.1.15, gadB, gadA, GAD	99.09	0
AHL	K11752	ribD	98.96	0
AHL	K20530	trbE	45.76	5.75×10^{-06}
AHL	K20266	trbJ	43.43	9.60×10^{-66}
AHL	K15850	luxN	42.50	1.47×10^{-16}
AHL	K18139	oprM	41.46	7.49×10^{-29}
AHL	K20527	trbB	41.10	5.62×10^{-06}
AHL,AI-2,Others	K10912	luxO	50.22	4.70×10^{-68}
AHL,DSF	K20276	bapA	67.57	3.25×10^{-130}
AI-2	K07173	luxS	100.00	1.73×10^{-126}
AI-2	K10909	luxQ	43.86	1.07×10^{-15}
AIP	K03100	lepB	99.67	0
cdiGMP	K13590	DGC	100.00	0
cdiGMP	K13069	E2.7.7.65	99.51	0
cdiGMP	K21023	mucR	98.96	3.24×10^{-110}
cdiGMP	K13243	dos	75.47	5.08×10^{-14}
cdiGMP	K21021	tpbB	48.77	3.38×10^{-37}
cdiGMP	K20963	acgA	48.32	5.41×10^{-71}
cdiGMP	K21024	bifA	47.34	8.80×10^{-46}
cdiGMP	K21019	sadC	46.15	7.61×10^{-38}
cdiGMP	K21084	yegE	45.93	3.30×10^{-39}
cdiGMP	K21090	adrB	42.31	2.77×10^{-56}
PQS	K06193	phnA	100.00	2.14×10^{-138}
PQS	K01657	trpE	97.97	0
PQS	K01658	trpG	97.44	1.17×10^{-139}
DSF	K10914	crp	100.00	5.11×10^{-158}
DSF	K01897	ACSL, fadD	99.82	0

DSF	K21688	rpfB	57.69	1.38×10^{-20}
DSF	K10715	rpfC	47.28	3.02×10^{-55}
DSF	K13815	rpfG	44.88	8.04×10^{-27}
DSF,Others	K13816	rpfF	42.68	8.29×10^{-09}
Others	K07667	kdpE	100.00	4.90×10^{-166}
Others	K07666	qseB	100.00	1.29×10^{-161}
Others	K03210	yajC	100.00	9.52×10^{-76}
Others	K03075	secG	100.00	7.37×10^{-76}
Others	K03073	secE	100.00	1.44×10^{-82}
Others	K03071	secB	100.00	1.12×10^{-116}
Others	K03217	yidC, spoIIIJ, OXA1, ccfA	100.00	0
Others	K11749	resP	100.00	0
Others	K03106	SRP54	100.00	0
Others	K03110	ftsY	100.00	0
Others	K03070	secA	99.89	0
Others	K14645	K14645	99.88	0
Others	K03076	secY	99.78	0
Others	K07645	qseC	99.57	0
Others	K02055	ABC.SP.S	60.29	1.30×10^{-156}
Others	K02052	ABC.SP.A	56.36	2.44×10^{-85}
Others	K07715	glrR	55.81	6.47×10^{-77}
Others	K07692	degU	53.57	1.54×10^{-11}
Others	K20488	nisR	50.00	8.37×10^{-50}
Others	K02031	ddpD	47.76	5.23×10^{-06}
Others	K02032	ddpF	47.09	4.15×10^{-68}
Others	K01996	livF	46.48	1.44×10^{-07}
Others	K12257	secDF	46.39	7.67×10^{-68}
Others	K20344	blpA	45.68	6.57×10^{-62}
Others	K01999	livK	45.61	2.14×10^{-06}
Others	K02490	spo0F	45.46	1.01×10^{-09}
Others	K02034	ABC.PE.P1	45.11	2.19×10^{-80}
Others	K03317	TC.CNT	44.13	7.21×10^{-118}
Others	K02054	ABC.SP.P1	43.89	1.82×10^{-43}
Others	K20490	nisF	43.00	7.55×10^{-42}
Others	K02035	ABC.PE.S	42.92	1.92×10^{-60}
Others	K14982	ciaH	42.11	4.21×10^{-13}
Others	K02053	ABC.SP.P	41.51	6.68×10^{-20}

Others	K02033	ABC.PE.P	40.85	1.30×10^{-57}
Others	K15583	oppD	40.56	1.49×10^{-71}
